# SGLT2 Inhibitors Empagliflozin and Canagliflozin Ameliorate Allergic Asthma Responses in Mice

**DOI:** 10.3390/ijms25147567

**Published:** 2024-07-10

**Authors:** Ye-Eul Lee, Dong-Soon Im

**Affiliations:** Department of Fundamental Pharmaceutical Sciences, Graduate School, Kyung Hee University, Seoul 02446, Republic of Korea; eul08@naver.com

**Keywords:** asthma, allergy, canagliflozin, SGLT2, pulmonary pharmacology

## Abstract

Inhibitors of sodium/glucose cotransporter 2 (SGLT2), such as empagliflozin and canagliflozin, have been widely used to block glucose reabsorption in the proximal tubules of kidneys in patients with diabetes. A meta-analysis suggested that SGLT2 inhibitors are associated with a decreased risk of asthma development. Therefore, we investigated whether SGLT2 inhibitors could suppress allergic asthma. Empagliflozin and canagliflozin suppressed the in vitro degranulation reaction induced by antigens in a concentration-dependent manner in RBL-2H3 mast cells. Empagliflozin and canagliflozin were administered to BALB/c mice sensitized to ovalbumin (OVA). The administration of empagliflozin or canagliflozin significantly suppressed OVA-induced airway hyper-responsiveness and increased the number of immune cells and pro-inflammatory cytokine mRNA expression levels in bronchoalveolar lavage fluid. The administration of empagliflozin and canagliflozin also suppressed OVA-induced histopathological changes in the lungs. Empagliflozin and canagliflozin also suppressed serum IgE levels. These results suggested that empagliflozin and canagliflozin may be applicable for the treatment of allergic asthma by suppressing immune responses.

## 1. Introduction

Bronchial asthma is characterized by airway hyper-responsiveness, mucosal inflammation in the airway, and reversible obstruction of airflow [1,2]. Historically, drug development for asthma has been focused on agents that promote the dilation of smooth muscles in the bronchial airways such as salmeterol and vilanterol, agonists of β_2_ adrenergic receptors, or inhibit the contraction of smooth muscles such as ipratropium and umeclidinium, antagonists of muscarinic acetylcholine receptors [3,4,5]. On the other hand, anti-inflammatory or immunosuppressive drugs such as inhaled corticosteroids, montelukast (leukotriene D_4_ receptor antagonist), and zileuton (5-lipoxygenase inhibitor) have been clinically applied [4,6,7]. In recent years, several biologic antibodies have been clinically introduced such as omalizumab (anti-IgE), dupilumab (anti-IL-4), and mepolizumab (anti-IL-5), because IgE and Th2 cytokines (IL-4, IL-5, and IL-13) play crucial roles in allergic asthma pathogenesis [8,9,10,11]. We have been searching for anti-inflammatory or immunosuppressive drugs for asthma treatment from natural resources such as alisol B 23-acetate [12] and synthetic chemicals including selonsertib (inhibitor of apoptosis signal-regulating kinase 1), elafibranor (dual agonist of peroxisome proliferator-activated receptor α and δ), NJK14047 (inhibitor of p38 mitogen-activated protein kinase), and AR420626 (agonist of free fatty acid receptor 3) [13,14,15,16].

Adding new clinical applications of the marketed drugs, which is known as drug repositioning, is a fast way of new drug development because clinical safety, tolerability, and doses for the marketed drugs have been already established [17,18,19,20]. Type 2 diabetes mellitus is a metabolic disorder characterized by insulin resistance, which is caused by chronic low-grade inflammation in the adipose tissues [21]. We have investigated whether any therapeutics for type 2 diabetes mellitus had anti-inflammatory or immunosuppressive effects, and found that inhibitors of sodium/glucose cotransporter 2 (SGLT2), such as canagliflozin and empagliflozin, might be applicable for allergic asthma because they protect against cardiovascular disorders and renal injury, and reduce cardiovascular mortality in diabetic patients [22,23,24,25,26,27,28,29]. In addition, SGLT2 inhibitors ameliorate atherosclerosis development in non-diabetic *ApoE*-gene-deficient mice and suppress the serum concentrations of inflammatory cytokines [23,27]. This strongly implies that they may have anti-inflammatory effects. Actually, the inhibitors of SGLT2 have been shown to decrease the levels of NF-κB, CCL2, and IL-6, indicating anti-inflammatory and anti-fibrotic effects in the kidney tissues of animals [25,29,30,31]. Similarly, inflammatory parameters, that is, TNF-α, IL-1β, MCP-1, IL-6, and CRP levels, have been improved by treatment of SGLT2 inhibitors in mice [30]. Canagliflozin has also been reported to be an outstanding anti-inflammatory drug, among other SGLT2 inhibitors, used in lipopolysaccharide-treated mice in vivo and in human immune cells in vitro [32,33]. Canagliflozin suppresses the levels of pro-inflammatory cytokines, such as TNF-α, IL-1β, and IL-6, and balances the proportions of M1 and M2 macrophages beneficially in vivo [22]. In addition, canagliflozin administration reduces IL-6 levels in human serum [32]. Furthermore, a meta-analysis suggested that SGLT2 inhibitors are associated with a decreased risk of developing asthma [34]. Therefore, we hypothesized that SGLT2 inhibitors could suppress allergic asthma by suppressing inflammatory immune responses, and investigated it using an ovalbumin (OVA)-induced allergic asthma model in BALB/c mice.

## 2. Results

### 2.1. Empagliflozin and Canagliflozin Suppresses Mast Cell Degranulation

**Figure 1:**
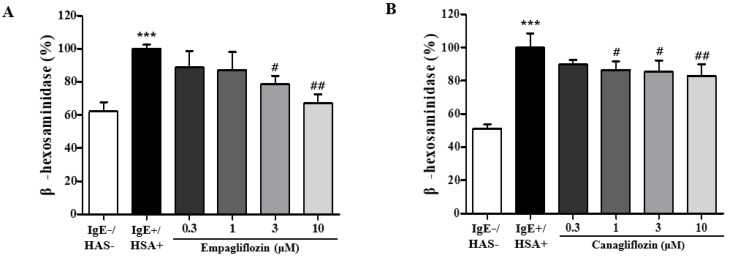
Effects of empagliflozin and canagliflozin on the degranulation of RBL-2H3 mast cells. RBL-2H3 cells were stimulated with dinitrophenyl-human serum albumin (DNP-HSA) after sensitization with anti-dinitrophenyl immunoglobulin E (DNP-IgE) for 18 h. Empagliflozin (**A**) and canagliflozin (**B**) were added at the concentrations indicated 30 min before DNP-HSA stimulation. There was basal degranulation observed in negative control samples without DNP-HSA and DNP-IgE treatment, and the degranulation seen with DNP-HAS and DNP-IgE was used as a positive control. The results are shown as means ± the standard error of the mean (SEM, *n* = 3). *** *p* < 0.001 vs. the HSA-untreated group. # *p* < 0.05, ## *p* < 0.01 vs. the HSA-treated group.

Among the SGLT2 inhibitors, we chose empagliflozin and canagliflozin based on previous studies showing that they inhibited chronic inflammation, including atherosclerosis. We tested whether empagliflozin and canagliflozin could suppress the human serum albumin (HSA, antigen)-induced degranulation of mast cells. The degranulation reaction was induced by HSA exposure in RBL-2H3 mast cells. The activity of β-hexosaminidase in the medium was assessed as a parameter of mast cell degranulation. Treatment with empagliflozin or canagliflozin significantly inhibited the HSA-induced increase in β-hexosaminidase activity in the medium at doses of 3 and 10 μM for empagliflozin (Figure 1A) and 1~10 μM for canagliflozin (Figure 1B). MTT assays were applied to measure cytotoxicity, but there was no significant change observed from concentrations of both SGLT2 inhibitors up to 30 μM in RBL-2H3 cells.

### 2.2. Empagliflozin and Canagliflozin Suppressed Ovalbumin-Induced Airway Hyper-Responsiveness in Mice

**Figure 2:**
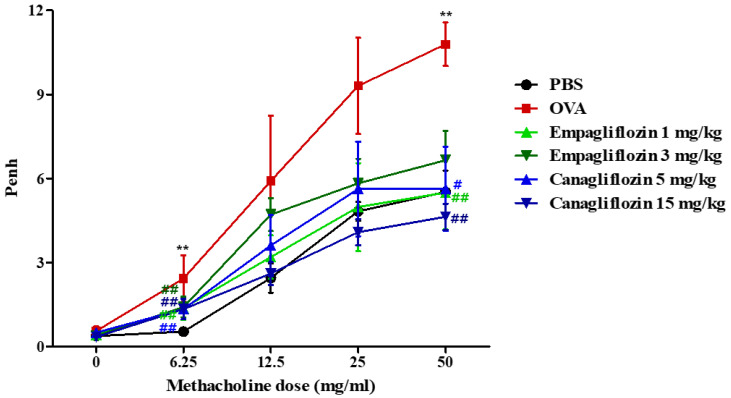
Effects of empagliflozin and canagliflozin on airway hyper-responsiveness in an OVA-induced murine asthma model. We determined Penh (enhanced pause) values as airway hyper-responsiveness in empagliflozin (1 or 3 mg/kg)-, canagliflozin (5 or 15 mg/kg)-, or PBS-treated mice by rising the methacholine concentrations. PBS: phosphate-buffered saline (PBS)-treated mice, OVA: ovalbumin (OVA)-challenged mice, OVA + empagliflozin (1 mg/kg), OVA + empagliflozin (3 mg/kg), OVA + canagliflozin (5 mg/kg), and OVA + canagliflozin (15 mg/kg). The results are shown as means ± the SEM (*n* = 5). ** *p* < 0.01 vs. the PBS-treated group, ## *p* < 0.01, # *p* < 0.05 vs. the OVA-treated group.

The in vitro suppression of degranulation by empagliflozin and canagliflozin was further investigated in OVA-induced allergic asthma. We selected two doses (1 and 3 mg/kg) of empagliflozin for intraperitoneal injection, because orally 10 mg/kg empagliflozin has been used in rodent experiments [25,26,28,29]. In the case of canagliflozin, 50 mg/kg canagliflozin has been used orally, which is five times higher than the value for empagliflozin [22,35]. Therefore, we selected two doses (5 and 15 mg/kg) of canagliflozin [22,35]. Airway hyper-responsiveness was determined using enhanced pause (Penh) values, as determined using a plethysmometer. The OVA induction of allergic asthma resulted in elevated Penh values at dosages of 6.25 mg/mL and above, whereas the administration of empagliflozin and canagliflozin suppressed the elevated Penh values (Figure 2) to approximately the values in phosphate-buffered saline (PBS)-treated mice.

### 2.3. Empagliflozin and Canagliflozin Suppressed the OVA-Induced Increase in Immune Cell Counts and mRNA Expression Levels of Pro-Inflammatory Cytokines in Bronchoalveolar Lavage Fluid

**Figure 3:**
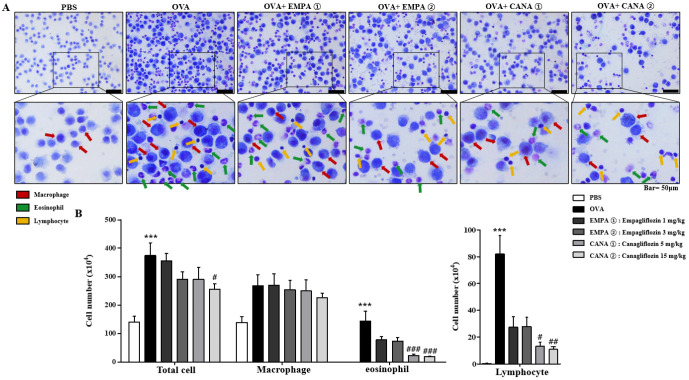
Effects of empagliflozin and canagliflozin on the rises in immune cell numbers induced by OVA in BALF. (**A**) Mice sensitized with OVA twice by intraperitoneal injection at day 0 and 14 were subsequently exposed to OVA nebulized on D28, D29, and D30. Empagliflozin (1 and 3 mg/kg) or canagliflozin (5 and 15 mg/kg) was intraperitoneally injected 30 min before the challenge of OVA. We stained and counted the cells in the bronchoalveolar lavage fluid (BALF) using the May–Grünwald stain. Lined areas on upper panels are enlarged in lower panels. Red arrows indicate macrophages, green arrows indicate eosinophils, and yellow arrows indicate lymphocytes. (**B**) Numbers of lymphocyte, eosinophil, macrophage, and total cells in the BALF are shown. The cell count results are shown as means ± the SEM (*n* = 5). *** *p* < 0.001 vs. the PBS-treated one, ### *p* < 0.001, # *p* < 0.05, ## *p* < 0.01 vs. the OVA-treated one.

We analyzed the number and distribution of immune cells in bronchoalveolar lavage fluid (BALF). The numbers of lymphocytes, eosinophils, macrophages, and total cells were higher in mice treated with OVA than PBS (Figure 3A,B). Canagliflozin significantly reduced the rise in the counts of lymphocytes, eosinophils, and total cells induced by OVA, but not macrophages (Figure 3A,B). Empagliflozin showed the suppression, but not significantly (Figure 3A,B).

**Figure 4:**
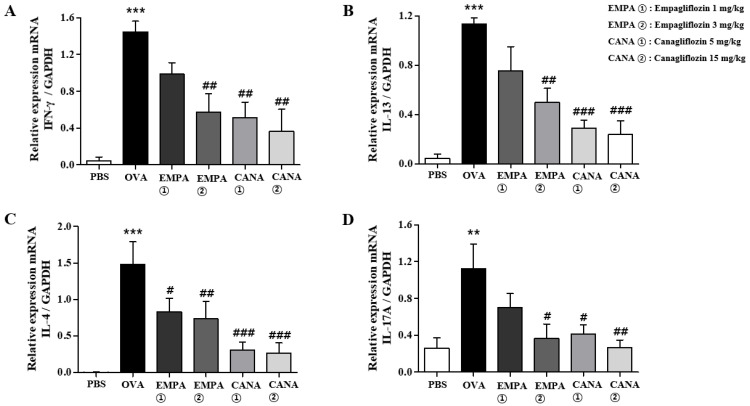
Effects of empagliflozin and canagliflozin on the expression levels of cytokines mRNAs in BALF cells. The levels of the Th2 cytokines Il-4 and Il-13, the Th1 cytokine Ifn-γ, and the Th17 cytokine Il-17a mRNA expression of in BALF cells were determined. (**A**) Ifn-γ, (**B**) Il-13, (**C**) Il-4, and (**D**) Il-17a. The levels of cytokines mRNAs were expressed relative to glyceraldehyde-3-phosphate dehydrogenase (GAPDH), a housekeeping gene. Values are shown as means ± the SEM (*n* = 5). ** *p* < 0.01, *** *p* < 0.001 vs. the PBS-treated one, ### *p* < 0.001, # *p* < 0.05, ## *p* < 0.01 vs. the OVA-treated one.

The levels of pro-inflammatory cytokines mRNA expression in BALF cells were determined using a quantitative reverse transcription polymerase chain reaction. It has been shown that Th2 cytokines of IL-13, Il-4, and IL-5 are deeply involved in the early phase of allergic asthma pathogenesis [36,37]. However, in the chronic phase, Th1 and Th17 cytokines have been found to be involved in the allergic asthma pathogenesis [38,39,40]. Therefore, we measured not only Il-4 and Il-13 (Th2 cytokines) but also Inf-γ (Th1 cytokine) and Il-17a (Th17 cytokine) mRNAs in BALF cells. OVA-induced allergic asthma increased the levels of Inf-γ (Th1 cytokine), Il-4 and Il-13 (Th2 cytokines), and Il-17a (Th17 cytokine) mRNAs in BALF cells, whereas the administration of empagliflozin or canagliflozin administration reduced the extent of these increases (Figure 4).

### 2.4. Empagliflozin and Canagliflozin Suppressed Histopathological Changes in the Airways

**Figure 5:**
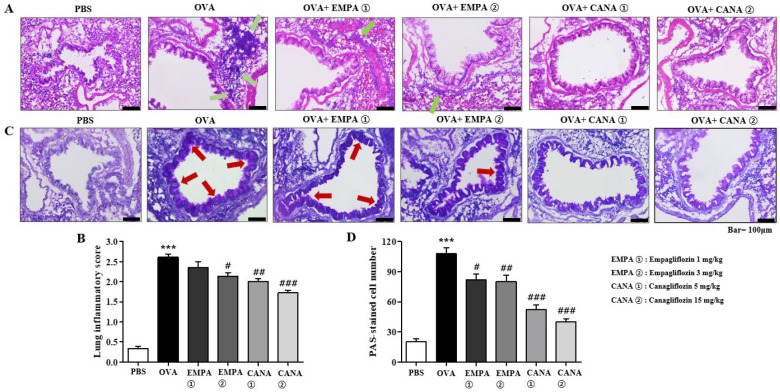
Effects of empagliflozin and canagliflozin on airway inflammation and mucin production. (**A**) Hematoxylin and eosin (H&E)-stained sections of lung tissues from the PBS-, OVA-, empagliflozin (1 or 3 mg/kg)-, and canagliflozin (5 or 15 mg/kg)-treated groups. The small navy-blue dots around the bronchioles are eosinophils. In the PBS-treated group, we rarely observed eosinophils. In the OVA-treated group, we found extensively accumulated eosinophils around the bronchioles (green arrows). (**B**) A histogram of the inflammatory scores in H&E-stained slides. (**C**) Periodic acid–Schiff (PAS)/hematoxylin-stained sections of lung tissues from the PBS-, OVA-, empagliflozin (1 or 3 mg/kg)-, and canagliflozin (5 or 15 mg/kg)-treated groups. Mucin is stained purple with PAS. In the OVA-treated group, a darker and thicker purple color was observed surrounding the bronchioles compared with that in the PBS-treated group (red arrows). (**D**) A histogram of the numbers of PAS-stained cells in the slides. Values represent means ± the SEM (*n* = 5). *** *p* < 0.001 vs. the PBS-treated one, ### *p* < 0.001, # *p* < 0.05, ## *p* < 0.01 vs. the OVA-treated one.

Histopathological analyses were conducted by using two different staining techniques, that is, periodic acid–Schiff (PAS) and hematoxylin and eosin (H&E) staining. H&E staining of lung samples was used to see the degree of airway inflammation. It showed the typical phenotypes of allergic asthma, that is, the accumulation of immune cells around the peribronchial areas. Severe inflammation was indicated by peribronchial infiltration of eosinophils in OVA-treated mice (Figure 5A). The number of eosinophils was reduced in empagliflozin- or canagliflozin-treated mice (Figure 5A). The intensity of inflammation was determined semi-quantitatively and is shown as a histogram in Figure 5B, which indicates a significant increase after OVA treatment and suppression by the treatments of empagliflozin and canagliflozin (Figure 5B). The average degree of inflammation was approximately 2.6 in the OVA-treated group, and empagliflozin and canagliflozin significantly reversed it (Figure 5B).

PAS staining of lung samples was used to see the degree of mucin hypersecretion, a typical feature of allergic asthma. In the PAS-stained samples from OVA-treated mice, the areas surrounding the bronchioles were stained with a darker violet color than those in the PBS-treated mice, implying hypersecretion of mucin (Figure 5C). However, the treatment of empagliflozin or canagliflozin significantly reduced the darkness and thickness of the violet-colored areas, implying there was less mucin production (Figure 5C). We counted the number of PAS-positive cells around the bronchioles and have presented them as histograms (Figure 5D). The number of PAS-positive cells was higher in the group treated with OVA rather than PBS (110 vs. 20; Figure 5D). The treatment of empagliflozin or canagliflozin significantly suppressed these effects (Figure 5D).

### 2.5. Empagliflozin and Canagliflozin Suppressed the OVA-Induced Increase in Expression Levels of Pro-Inflammatory Cytokines mRNAs in the Lungs

**Figure 6:**
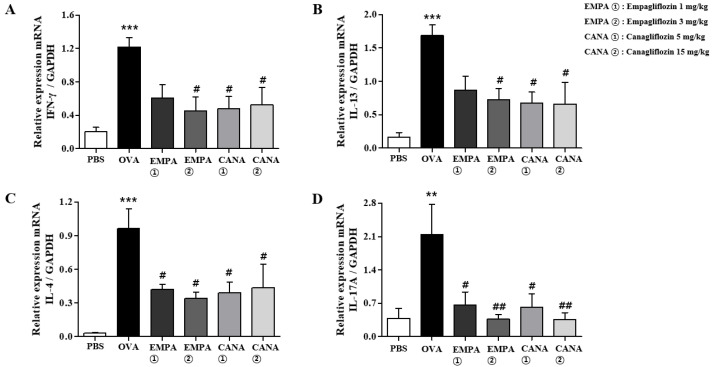
Effects of empagliflozin and canagliflozin on the expression levels of cytokines mRNAs in the lungs. The expression of the Th1 cytokine Ifn-γ, the Th2 cytokines Il-4 and Il-13, and the Th17 cytokine Il-17a mRNAs in the lung tissues were determined. (**A**) Ifn-γ, (**B**) Il-13, (**C**) Il-4, and (**D**) Il-17a. The levels of cytokines mRNAs were expressed relative to glyceraldehyde-3-phosphate dehydrogenase (GAPDH), a housekeeping gene. Values are shown as means ± the SEM (*n* = 5). *** *p* < 0.001, ** *p* < 0.01 vs. the PBS-treated one, # *p* < 0.05, ## *p* < 0.01, vs. the OVA-treated one.

We also assessed the expression levels of Il-4, Il-13, Inf-γ, and Il-17a mRNAs in the lungs. The levels of these cytokines mRNAs were increased in the lungs of OVA-treated mice, and the treatment of empagliflozin or canagliflozin significantly reduced this increase (Figure 6).

### 2.6. Empagliflozin and Canagliflozin Suppressed the Increase in Serum IgE Levels and BALF IL-13 Levels Induced by OVA

**Figure 7:**
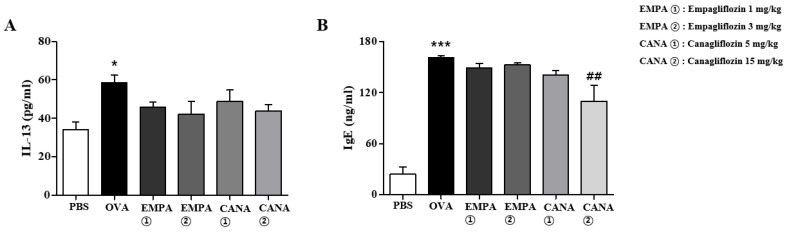
Effects of empagliflozin and canagliflozin on IL-13 levels in BALF and IgE levels in serum. IL-13 levels in BALF (**A**) and IgE protein levels in serum (**B**) were measured by enzyme-linked immunosorbent assays. The results are shown as means ± the SEM (*n* = 5). *** *p* < 0.001, * *p* < 0.05 vs. the PBS-treated one, ## *p* < 0.01 vs. the OVA-treated one.

Given that the mRNA levels of Th2 cytokines increased, the protein levels of Il-13 were assessed using an enzyme-linked immunosorbent assay. The Il-13 levels were significantly higher in the BALF from mice treated with OVA rather than PBS (Figure 7A). The treatment of empagliflozin or canagliflozin suppressed this increase; however, the effect was not significant (Figure 7A). Next, we assessed serum IgE levels. IgE levels in the serum of OVA-treated mice were significantly higher than those in PBS-treated mice (Figure 7B). Canagliflozin treatment significantly suppressed this increase, but not empagliflozin (Figure 7B).

## 3. Discussion

We demonstrated that SGLT2 inhibitors, empagliflozin and canagliflozin, suppressed HSA-induced mast cell degranulation in vitro in a concentration-dependent manner and showed their therapeutic efficacy against OVA-induced allergic asthma in vivo. Mechanistically, how did SGLT2 inhibitors suppress in vivo OVA-induced allergic asthma? Given that antigen-induced asthmatic attacks are evoked by mast cell degranulation [41], the suppressive effects of SGLT2 inhibitors on mast cell degranulation might be an explanation partly for their in vivo efficacy. However, how did SGLT2 inhibitors suppress the mast cell degranulation? Because the expression of SGLT2 is limited to the renal proximal tubule [42,43] and it was not detectable in RBL-2H3 cells and in immune organs [23]; the inhibition of SGLT2 may not be a possible mechanism in the mast cells. Considering that the chronic phase of allergic asthma was made by combined inflammatory responses of Th1, Th2, and Th17 cells and their cytokines [36,37,38,39,40], the anti-inflammatory effects of SGLT2 inhibitors may be an explanation mainly for the in vivo efficacy of SGLT2 inhibitors. In fact, we observed strong evidence supporting anti-inflammatory and immunosuppressive effects of SGLT2 inhibitors; (1) the suppression of immune cell accumulation in the BALF, (2) the suppression of inflammatory cytokine levels in the BALF and the lung, (3) the suppression of mucin hypersecretion and histopathological changes, and (4) reduced levels of IgEs. These results are consistent with the previous observation that SGLT2 inhibitors suppresses the levels of pro-inflammatory cytokines, such as TNF-α, IL-1β, and IL-6, and balances the proportions of M1 and M2 macrophages beneficially in vivo [22,25].

Although there have been multiple observations that SGLT2 inhibitors showed anti-inflammatory results, such as atherosclerosis, myocardial infarction, and renal injury [25,26,27,28], SGLT2 inhibition has not been proposed as a cause of the anti-inflammatory effects of another SGLT2 inhibitor, dapagliflozin [23]. Furthermore, SGLT2 is highly expressed in the kidneys, but not in other organs in mice [33]. Therefore, it is reasonable to assume that the suppressive effects of SGLT2 inhibitors on allergic asthma are not mediated by the inhibition of SGLT2 in immune cells. Then, how could SGLT2 inhibitors show anti-inflammatory effects in the lungs and immune cells in the BALFs in the present study and the previous other cardiovascular disease models [25,26,27,28]? Presently, we are not able to pin-point the direct targets of SGLT2 inhibitors in the anti-asthmatic efficacy. However, we could discuss and propose a possible mechanism. First of all, SGLT2 inhibitors may block glycolysis in immune cells because they have a common glucose structure, which is similar to 2-deoxy glucose [33]. As 2-deoxy glucose cannot be metabolized in the cytosol, SGLT2 inhibitors may bind to and inhibit intracellular enzymes for glucose metabolism, resulting in an energy deficient state in the immune cells [33]. Actually, canagliflozin inhibits intracellular glycolysis, which promotes p62-mediated IL-1 degradation and enhances autophagy [33]. Consequently, the energy deficient state may increase the cytosolic concentration of AMP, which augments the phosphorylation of AMP-activated protein kinase (AMPK). AMPK action may indirectly suppress the inflammatory factor NF-κB, thereby resulting in the polarization of macrophages to an anti-inflammatory M2 phenotype [23]. This possibility is based on multiple observations by SGLT2 inhibitors: (1) canagliflozin inhibits glycolysis and enhances autophagy [33]; (2) empagliflozin augments AMPK phosphorylation, which induces M2 macrophages within the adipose tissues and liver [44]; (3) dapagliflozin polarizes cardiac macrophages to the anti-inflammatory M2 phenotype [23]; and (4) the activation of AMPK suppresses the expression of pro-inflammatory cytokines by the downregulation of NF-κB signaling in murine macrophages [45]. Therefore, we are sure SGLT2 is not the direct target of the efficacy, and we propose a possible explanation of how SGLT2 inhibitors show anti-allergic asthma efficacy as described above. Further investigations are required to establish a possible mechanism.

As we proposed in the Introduction, drug repositioning is a fast way to develop new therapeutics for allergic asthma. The present results may be a starting point to reposition SGLT2 inhibitors to anti-asthmatic drugs, in addition to their anti-diabetic efficacy.

## 4. Materials and Methods

### 4.1. Materials

We purchased empagliflozin and canagliflozin from MedchemExpress (Cat no. BI 10730 and HY-10451, respectively, Purity 99.80%, Monmouth Junction, NJ, USA). Alum and OVA were obtained from Sigma-Aldrich (St. Louis, MO, USA).

### 4.2. RBL-2H3 Mast Cells

We obtained rat RBL-2H3 mast cells from the American Type Culture Collection (ATCC, Manassas, VA, USA). We cultured RBL-2H3 cells in high glucose Dulbecco’s modified Eagle medium (DMEM) containing 10% (*v*/*v*) heat-inactivated fetal bovine serum along with 2 mM glutamine, 100 U/mL penicillin, 1 mM sodium pyruvate, and 50 μg/mL streptomycin was placed in a humidified incubator at 37 °C in 5% CO_2_ [14].

### 4.3. Determination of Mast Cell Degranulation

We determined the degranulation of RBL-2H3 cells by assessing the β-hexosaminidase activity in the media. We used mouse monoclonal anti-dinitrophenyl immunoglobulin E (DNP-IgE) and human DNP albumin to stimulate degranulation [14].

### 4.4. BALB/c Mice

We obtained five-week-old female BALB/c mice from DBL (Seoul, Republic of Korea). The mice were housed in a laboratory animal facility at Kyung Hee University and provided water and food ad libitum. The Institutional Animal Care Committee of the university reviewed and approved the study protocol (Approval Number, KHSASP-23-493).

### 4.5. OVA-Induced Asthma Model and Canagliflozin Treatment

We divided female BALB/c mice aged six weeks into four groups (*n* = 5): a PBS-treated control one, an OVA-treated asthma one, an OVA-treated asthma one co-treated with canagliflozin (5 mg/kg), and an OVA-treated asthma one co-treated with canagliflozin (15 mg/kg). We injected 1 mg aluminum hydroxide and 50 μg OVA intraperitoneally to sensitize the mice twice on day 0 (D0) and D14. We exposed mice for 30 min to OVA nebulized (1% OVA or PBS) by an ultrasonic nebulizer (Philips) on D28, D29, and D30. We injected canagliflozin intraperitoneally 30 min before exposure to OVA (D28, D29, and D30). We collected bronchoalveolar lavage fluid (BALF) on D32 from the lungs, stained BALF cells, and analyzed the number of each cell type [13].

### 4.6. BALF Cell Counting and Analysis

We attached the immune cells of the BALF to a glass slide by centrifuging it with Cellspin^®^ (Hanil Electric, Seoul, Republic of Korea). Later, we fixed the cells in MeOH for 30 s, and stained them by incubating them in May–Grünwald solution for 8 min and subsequently in Giemsa solution for 12 min.

### 4.7. Measuring Airway Hyper-Responsiveness to Methacholine

We determined airway hyper-responsiveness on day 31 using PLY-UNR-MS2 (EMKA Technologies, Paris, France), a non-invasive measurement of lung function. We placed the mice in the chamber of a barometric plethysmograph, recorded the baseline for 3 min, and calculated the enhanced pause (Penh). We expressed the results as the increase in percentage in Penh following the challenge with increasing methacholine concentrations (0, 6.25, 12.5, 25, and 50 mg/mL) [13].

### 4.8. Histopathological Analysis of the Lung

We prepared lung tissue sections and stained them with hematoxylin and eosin (H&E) to assess immune cell infiltration, or with periodic acid-Schiff (PAS) to determine mucus-producing cells [13]. The degree of lung inflammation on a subjective scale of 0–3 was measured by a treatment-blind observer. We counted mucin-secreting cells stained with PAS around the bronchioles in two lung sections of a mouse, and expressed mucous production as the number of PAS-positive cells of bronchiole per millimeter of the length of bronchial basal lamina after measurement with ImageJ software version 1.54 (National Institute of Health, Bethesda, MD, USA) [13].

### 4.9. Measurement of the Protein Levels of IgE in Serum and IL-13 in BALF

We used ELISA kits (eBioscience, San Diego, CA, USA) to determine the protein levels of IgE in serum and IL-13 in BALF of the mice. We obtained capture antibodies and biotinylated detection antibodies specific for IL-13 from eBioscience (Cat no. 14-7043-68 and 33-7135-68B) and a mouse IgE-uncoated ELISA kit for IgEs from Thermo Fisher (Cat no. 88-50460-88, Waltham, MA, USA). We measured the absorbance at 450 nm [13].

### 4.10. Statistics

We performed statistical analyses using GraphPad Prism software version 5.0 (GraphPad Software, Inc., La Jolla, CA, USA). We used one-way analysis of variance (ANOVA), followed by Tukey’s multiple comparison test to compare the differences and significance among multiple groups. We expressed data as means ± standard error of the mean (SEM). We considered differences statistically significant at *p* < 0.05.

## 5. Conclusions

The present findings suggest that SGLT2 inhibitors may be useful drugs for treating allergic asthma by drug repositioning.

## Data Availability

Data are available upon request.
